# The Therapeutics of Parturition

**Published:** 1898-04

**Authors:** C. E. Ide

**Affiliations:** Chicago, Ill.


### THE THERAPEUTICS OF PARTURITION.

By C. E. IDE, M. D., Chicago, Ill.

It is not intended to enter upon a lengthy consideration of this subject, but to call especial attention to certain points which experience has taught are frequently neglected or unknown.

The therapeutics of labor really includes the therapeutics of pregnancy; for all through the latter state we look forward to the former, and to those means which we adopt to assist the parturient woman we should add those which we employ in preparing the pregnant woman for the coming siege.

We will consider, then, that our subject includes prophylaxis, as well as a curative and sustaining treatment during parturition itself. In addition to this, we would call attention to the use of anesthetics in labor and to puerperal convalescence.

### PROPHYLACTIC TREATMENT DURING PREGNANCY.

There are many annoying conditions which may arise during pregnancy to rob prospective motherhood of its joy and satisfaction, and vest it with an aspect as terrible as that of disease itself. These we would seek to remove as simply and expeditiously as possible, one by one, as they appear, not allowing them to accumulate until our patient is worn out and in a serious condition. In fact, it is well to forestall these annoyances, and institute, by the end of the third month of pregnancy, such mild and simple, though accurate and sufficient, treatment as will make the condition a comfortable and normal one, and stimulate vital functions. And we can believe that, while we thus benefit the mother, we also surround her child with those conditions which conduce to normal, perfect development and function. In addition to this, we should watch the urine, not depending upon the presence or absence of albumen alone, but considering quantity and contents, and, not stopping here, make a microscopic examination once a week. This will avert many an unfortunate accident, and give us mental comfort and satisfaction.

These being our conclusions, how shall we go to work to fulfill them? Not, surely, by seeking some panacea which will, once for all, remove all chance of the appearance of uncomfortable conditions; not, indeed, by attempting to find one line of treatment powerful to cure or prevent all; not by becoming disgusted or discouraged and giving up altogether. No. But, believing that in quietness and confidence shall be our strength, by directing to each untoward condition which arises its own antagonist, provided for us by the constancy and assiduity of faithful minds and hands.

To take up certain conditions in detail, and consider their proper treatment: How shall we make pregnancy comfortable and normal, and stimulate vital functions? Why, by providing the cells and organs by which those functions are performed with food and tonics. We can prescribe, for instance (especially during the last three months of pregnancy), strychnine arseniate, gr. $\frac{1}{134}$, every four hours, as a vital incitant, and iron arseniate, gr. $\frac{1}{6}$, three to six times a day, or iron lemonade, to replace the waste of albumen and blood corpuscles; and these dosimetrically, that is, in small doses frequently repeated, that the desired physiologic effect only may be attained, and no overstimulation or exhaustion, the very opposite of what we desire, produced.

From the fourth to the sixth or seventh month, the time of most rapid development in the bony framework of the fetus, if we provide the fetus with some form of lime, through the mother's circulation, there will be so much the less drain upon her tissue. We can do this, and provide some phosphorus at the same time, by administering the glycerophosphate of calcium, gr. 3 to 5, three times a day. Another means of providing phosphorus is by the administration of phosphoric acid lemonade, gr. $\frac{1}{6}$, dissolved in water, several times a day, for the thirst which sometimes all but consumes the woman.

For the *irritability of the bladder*, which is often so very annoying in the early weeks of pregnancy, nothing is better than benzoic acid or benzoate of lithium, gr. $\frac{1}{2}$, in water, frequently. If necessary, some hyoscyamine, gr. $\frac{1}{250}$, every hour until the desired effect is produced may be added for sedative effect.

The number of remedies which have been recommended for the *reflex nausea*, which is so common that every woman expects her pregnancy to be characterized by it, proves to us that there are some cases which can not be relieved medicinally. For those which can there is nothing better than cerium or silver or zinc salts; with small doses of cocaine (hypodermically) added, if necessary.

*Constipation*, the common foe of womankind, is here universally present, except in unusual cases of well-regulated bowels, or those from which there are freqnent movements, the result of chronic disease. Constipation is frequently the cause of the nausea which it is sought to relieve with remedies directed to the stomach locally. Autointoxication results from this constipation, with headache, vertigo, ocular disturbance, foul tongue, tinnitus, dyspnea, palpitation, abdominal distress, pelvic pain, sciatic pain and dysuria, or frequent micturition. The best remedy for this, and one which will serve also to quiet and sweeten the stomach, is an effervescent saline laxative. It is harmless, acts promptly, is not unpleasant to take, and produces just what we want, a fluid movement which can readily pass through a compressed rectum and carry out with it those toxic products which often play a large part in the causation of hydra-headed eclampsia.

Anemonin, a camphor derived from pulsatilla, is administered with good effect, in dose of gr. $\frac{1}{134}$, every two to four hours, in the *flatulence* and *flushes* which prove so annoying, disturbing respiration and the heart, often provoking nausea and causing great abdominal distress. This agent also has special reference to the generative function, which it strengthens. If given in small doses there is no danger of its conducing to abortion.

*Pica*, the morbid craving for unnatural food, a condition which is not easily explained or done away with, is often successfully treated with hyoscyamine. In addition to this, the woman is to be allowed all forms of food or drink she craves which can with any reason be given her. I once heard Professor McLane exclaim: "Give her almost anything she craves, even beer."

For the woman who "feels as if she should fly" all the time, or at intervals, nothing is better than macrotin, in dose of gr. $\frac{1}{6}$ every two or three hours. This "braces up" the sympathetic nervous system and removes congestion from the various nerve centers. Its effect is increased if combined with strychnine.

*Dropsies*, arising from pregnancy are readily dissipated with apocynin, unless they be due to organic diseases of the heart, liver, or kidneys; in which case the best thing to do is to obtain a consultation, so as to share the responsibility with a brother practitioner, and induce labor, or, if earlier, produce abortion. A child born of a mother with such organic disease is not desirable to family, society or state. If the dropsy is coupled with vertigo and tinnitus, administer iron with a view to relieving coexisting anemia, or, better, hydremia (plethora); and then give a saline laxative to remove all toxins resulting from the constipation which usually accompanies both pregnancy and anemia. If the dizziness still persists, think of organic disease, in the absence of marked indigestion, or ocular disease.

As we remarked above, it is important to keep the urine constantly in mind, watching the excretion of solids by the kidneys with care. The kidneys of the pregnant woman are greatly overworked, as is evidenced by the permanent damage frequently done them at that time. We should assist them all we can, and to this end can well administer colchicine, gr. $\frac{1}{134}$, five or six times in twenty-four hours, which has a powerful action in increasing the solids in the urine, especially when combined with caffeine arseniate.

In the *fatigue*, *debility* and *somnolence* which we often meet toward the close of pregnancy, the heart should be sustained with the "dosimetric trinity" granule, which is composed of digitalin (Germanic), gr. $\frac{1}{67}$, aconitine (amorphous), gr. $\frac{1}{134}$, and strychnine arseniate, gr. $\frac{1}{134}$. It strengthens and slows the heart, without producing too great blood tension, quickens the circulation, stimulates nutrition and brightens the whole aspect of the case.

When we feel that some form of *inflammation* threatens, or there are *distended veins* in any portion of the body, the resulting congestion clogging the circulation, we cannot do better than employ the "defervescent compound" granules, composed of digitalin and aconitine, as in the above, with veratrine, gr. $\frac{1}{134}$, in place of the strychnine. This strengthens and quiets the heart, and, at the same time, "bleeds the patient into her own arteries," removing the surplus blood from the venous system.

Last, but not least, by any means, nuclein (Aulde), gr. $\frac{1}{3}$, every two or four hours, is an excellent reconstructive adapted to all the above conditions, and administered with the utmost propriety in every case where we wish to stimulate each cell to full performance of its function.

### REMEDIES IN LABOR.

So much for our prophylaxis. And, having been assiduous in our attentions through pregnancy, we should not fail amidst the perils of childbirth. We can do much to facilitate labor, and easily and simply too. When the pains are *cramp-like and distressing*, when nerve energy and muscular power fail, when the os uteri is still firm and fails to dilate in spite of rhythmic pains, and the woman is becoming exhausted and pleads: "Let me alone; let me die;" or when there is inertia, with no attempt at headway, just give the patient three granules of strychnine arseniate, gr. $\frac{1}{134}$, and of hyoscyamine, gr. $\frac{1}{250}$ (the dose should be large and fearless), and repeat in an hour if needed. After the granules have been dissolved on the tongue, the effect will be prompt and surprising. The pains will continue strong, regular and effective, from the well-known stimulating effect of the strychnine upon longitudinal muscle-fiber and the cerebro-spinal system; they will lose their cramp-like and distressing character, and the circular fibers of the cervix and lower segment of the uterus will relax, from the effect of the hyoscyamine on circular muscle-fiber; nerve energy will return; muscular power will come up to par, and the patient will feel refreshed, as after a sleep. Not the least of the good effect of this treatment is a quieting of the mind, together with the whole nervous system. Anxiety gives way to confidence, despair gives way to hopefulness, and if it be not a case of impacted head, the end will soon come.

An *anesthetic* is an important essential in the treatment of those accidents which are bound to occur, from time to time, in labor. Its use is to be entered upon fearlessly and boldly, wherever indicated. The anesthetic which is particularly indicated in the class of cases which we are considering is chloroform. It is safe, can be made as safe as ether, if we first inform ourselves as to the true cause of death in cases which succumb to its influence. The day of argument, *pro* and *con*, as to whether heart paralysis or repiratory paralysis is the cause of death in such cases has passed. We now know, without question, that the cause of death from chloroform is vasomotor paralysis; that is the chloroform paralyzes the vasomotor system so that the capillaries of the body dilate to the extent of containing so much blood that the vital centers are deprived of their necessary blood-supply, and so of their pabulum. Hence we lower the head and elevate the extremities, in collapse from chloroform, performing artificial respiration until sufficient blood has returned to the vital centers in the medulla.

Bearing in mind, then, the true cause of death from chloroform, we will set our minds at rest on the question of danger, and "push" the anesthetic when demanded, if we "brace" the vasomotor system with a hypodermic injection of the antidote (atropine, gr. $\frac{1}{134}$, to be repeated as necessary) previous to beginning the administration of the chloroform.

In the operations of forceps delivery, turning, replacing of funis, changing of position, etc., it is necessary to be confident and fearless, and push the chloroform to extremes; else that which we desire, thorough relaxation, is not attained.

With regard to application of forceps, turning and changing of presentation, my friend Dr. W. C. Abbott, of Chicago, has suggested to me a procedure which facilitates these greatly, is easily done and which brings the field of operations within reach and away from the bed and discharges. It consists in elevating the foot of the bed and placing the woman in the knee-chest position. It is a procedure often adopted in replacing the funis, I am aware; but I have not seen it recommended for the above operations. So I conclude that Dr. Abbott is perhaps the originator of this method, and I would give him the credit which he deserves, if such be the case. In this procedure the intestines and uterus, with its contents, sink away from the outlet of the pelvis, leaving room for introduction of forceps or hand, and obviating the obscuring of the region by discharges. Experience long ago proved its utility in replacing the funis.

*Puerperal convalescence* can be greatly assisted and accelerated by the administration of strychnine and ergotin, together with digitalin and quinine, three times a day for two or three weeks. These assist involution in uterine and heart muscle, and the energizing and contracting effect of the strychnine and ergotin upon the uterine muscle serve to close and seal more effectually any avenues by which infection might enter the circulation of lymphatics on the interior surface of the uterus. The digitalin improves vascular tone and stimulates the heart and assists the kidneys in the excretion of the effete matter resulting from the process of involution throughout the body. The quinine is a nerve tonic and serves to abort any chills and chilly sensations arising from sapremia, or even infection. It also has a supplementary action in stimulating contraction of uterine muscle-fibre.

These, then, are the points to which it was desired to call attention; and while we medical men are bewailing the fact that our profession has, in many quarters, been dragged down to the level of a trade, it behooves us to grasp every opportunity to arise above such level and regain the heights which once our beloved work occupied. We should seek the better way, be consistently scientific, and, with docility, learn the lessons which the extremes to which the professional pendulum has swung, from time to time, have taught us. There is chance for improvement in many directions, and one such chance is that of prophylaxis, symptomatic treatment, the grappling with conditions which arise before they amalgamate and develop into something serious. Furthermore, while we direct treatment to such conditions, it behooves us to be scrupulous in our selection of means, and employ such remedies as will do the desired work without producing, collaterally, any harm. This we can accomplish by the use of active principles, rather than "whole-drug" preparations. We wish to produce certain effects, and if we can do so *cito, tuto et jucunde*, so much the better.

Confidence is a great desideratum in the practice of therapeutics, and this quality is begotten of a thorough knowledge of the means which we adopt and the results which they produce, rather than a hope that certain results will be attained. Further than this, the practice of therapeutics is impossible without power to diagnosticate correctly.

To sum up, then, the lesson to which we would call attention, we will adopt for our watchword: "To recognize the indications, to fulfill them, and, in doing so, adopt those means which experience has proved to be the very best."—*Medical Council.*

---

Nurse—Johnnie, the stork has just brought you a little baby. Wouldn't you like to see your little brother?

Johnnie—Naw, but I'd like to see the stork.

---

Having read the directions on the box, "Take one every two hours till gone," she sent in haste to the prescriber to know if he meant her or the pills.

